# Evaluation of Stress Distribution and Displacement in the Affected Periodontium of Mandibular Incisors Using Utility Intrusion and Reverse Curve of Spee Arch Wires: A Finite Element Analysis

**DOI:** 10.7759/cureus.85473

**Published:** 2025-06-06

**Authors:** Vazrala Vamsi Krishna Reddy, Ghanta Sunil, Soma Balaji, Soorabathula Sonika Mani kiran, Konni Prudhvi, RSVM Raghu Ram

**Affiliations:** 1 Orthodontics and Dentofacial Orthopedics, GSL Dental College and Hospital, Rajamahendravaram, IND; 2 Dentistry, GSL Dental College and Hospital, Rajamahendravaram, IND

**Keywords:** bone loss, finite element analysis, finite element model, reverse curve of spee arch wire, stress and displacement, utility intrusion arch wire

## Abstract

Objective: This study aims to evaluate stress distribution and displacement in the affected periodontium of mandibular incisors with utility intrusion arch wire and reverse curve of Spee (RCS) arch wire using finite element analysis (FEA).

Materials and methods: A 3D finite element model (FEM) of the mandibular arch was created, simulating mandibular incisors with three variations of bone loss (0%, 33%, and 66%) and 0.022” x 0.028” slot MacLaughlin, Bennett, and Trevisi prescription brackets. Utility intrusion arch wire and RCS arch wire were modelled using 0.017” x 0.025 stainless steel and subjected to activation. Stress distribution and displacement were calculated and analyzed.

Results: Comparison between utility intrusion arch wire and RCS arch wire, using FEM for mandibular incisors with varying bone loss (0%, 33%, and 66%), revealed alteration in the stress distribution and displacement patterns. Increasing bone loss at mandibular incisors raised stress distribution while reducing displacement, suggesting labial tipping. The utility intrusion arch wire exhibited higher stress distribution than the RCS arch wire at all bone loss levels. The RCS arch wire caused labial tipping of the mandibular central incisors. In contrast, the utility intrusion arch wire minimized displacement across all mandibular incisors in FEMs despite variations in bone loss. The utility intrusion arch wire displayed better torque control, reducing labial tipping compared to the RCS arch wire, irrespective of bone loss levels.

Conclusions: Elevated bone loss in mandibular incisors resulted in increased stress distribution and decreased displacement, implying a tendency toward labial tipping. The utility intrusion arch wire consistently exhibited higher stress distribution but minimized displacement compared to the RCS arch wire, highlighting its potential for better torque control and reduced labial tipping, particularly with the progression of horizontal bone loss.

## Introduction

Adult patients with periodontal diseases often experience bone loss, tooth migration, elongation, and spacing. These positional changes occur when the balance between available periodontal support and the forces acting on the teeth is disrupted, particularly in the presence of plaque-associated gingival inflammation [1]. As gingival disease progresses, bone height gradually recedes, leading to bony defects that can be vertical, horizontal, or osseous wall defects (one-, two-, or three-wall). Horizontal bone loss, where the bone height decreases while the margin remains perpendicular to the tooth surface, is the most common form [2]. Wu et al. found that crater defects were the most common (26.5%), followed by circumferential defects (23.4%) and three-wall defects (20.08%) [3]. Persson et al. highlighted the contrast in treatment options, with vertical bone loss (7.8% prevalence) showing a 96.8% treatment success rate, while horizontal defects (92.3% prevalence) had only 3.2% treatment success [4]. Management of osseous defects through periodontal therapy stabilizes the periodontal condition [5]. Orthodontic treatment is often recommended if malocclusion results from periodontal issues, as it can help improve the health of the periodontium.

Mandibular incisors are more prone to bone loss due to their anatomical location, which can lead to migration from their normal position and result in malocclusions such as a deep bite, crowding, or spacing. This change in bone levels affects the center of resistance of the teeth, thereby impacting the mechanics of orthodontic treatment. According to Kuruthukulam and Patil, when a horizontal force is applied, the center of resistance is the point between the apex and the alveolar crest, where bodily movement occurs [6]. The center of resistance of single-rooted teeth is typically located 33-42% of the root length from the alveolar crest. The location of the center of resistance can vary depending on factors like root length, surrounding bone, tooth morphology, and tissue response to force [6,7]. Jo et al.'s study using finite element analysis (FEA) found the center of resistance of mandibular incisors to be 13.0 mm apical and 6.0 mm posterior. In contrast, for the six mandibular anterior teeth, it was 13.5 mm apical and 8.5 mm posterior. For the complete mandibular dentition, the center of resistance was 13.5 mm apical and 25.0 mm posterior to the incisal edge of the mandibular central incisors [8].

Pathological changes in bone are commonly observed in deep bite conditions, which require orthodontic treatment to prevent further damage to the periodontal status. A deep bite refers to an orthodontic condition where the upper front teeth overlap significantly over the lower front teeth, causing the point of force application to be located closer to the roots of the lower teeth. Correction of deep bite is accomplished using anchor bends, V-bends, utility intrusion arches, and reverse curve of Spee (RCS) arch wires. Among these, utility intrusion arch wires and RCS arch wires provide better torque control during intrusion mechanics as they engage more effectively into the bracket slot [9]. Rickett's method for overbite correction uses a utility intrusion arch wire made from 0.016" × 0.016" square-edged blue Elgiloy wire, providing precise control over axial incisor inclination and intrusion [10]. McFadden et al. found the utility intrusion arch wire effective for incisor intrusion but noted significant root shortening with low-force treatment (25 g). These changes may be influenced by the altered center of resistance and reduced bone levels in mandibular incisors [11]. The RCS technique is commonly used to level the Spee curve in deep bite patients, primarily causing posterior tooth extrusion. Available wire dimensions include 0.014-0.020" round wires and various rectangular options. However, they may lead to undesirable axial inclinations and incisor flaring [10]. AlQabandi et al. found no significant difference between round and rectangular wires. However, they observed mandibular incisor proclination linked to uncontrolled tipping, reduced intercanine width, and crowding, although only 41% of the variability could be explained [12].

The outcomes of these various arch wires in different clinical scenarios can be best interpreted using biomechanical analysis, primarily through the finite element model (FEM). FEM simulates the effects of orthodontic tools on teeth, bones, and soft tissues, enabling the creation of personalized treatment plans and minimizing potential complications. By dividing structures into elements, FEM analyzes shape changes in all directions, offering more detailed insights than conventional cephalometrics [6,13].

No previous reports have documented the stress distribution and displacement on the affected periodontium of mandibular incisors using utility intrusion arch wire and RCS arch wire. Therefore, this study is aimed at assessing the stress distribution and displacement in the affected periodontium of mandibular incisors with utility intrusion arch and RCS arch wires using FEM. The investigation focuses on two commonly used intrusion mechanics: the utility intrusion arch and the RCS arch wire. To achieve this, three levels of periodontal support were modeled to simulate varying degrees of bone loss: 0% (intact periodontium), 33%, and 66% bone loss. The study explicitly measures and compares the resulting stress patterns and tooth displacement across these periodontal conditions to understand the biomechanical impact of each intrusion technique on compromised periodontal structures.

## Materials and methods

The research was conducted at GSL Dental College and Hospital in Rajahmundry, Andhra Pradesh, India, to develop a 3D FEM to simulate the effects of varying degrees of bone loss on the mandibular four incisors. The process began by obtaining high-resolution CT scans of an adult dry skull’s mandibular arch, recorded in Digital Imaging and Communications in Medicine (DICOM) format. These CT scans provided detailed axial and coronal sections, from which the geometric model of the mandibular arch was created. Using Catia V5 software (Dassault Systèmes, Vélizy-Villacoublay, France), the 3D model was meticulously built, capturing the shapes and curves of the teeth, periodontal ligament, and alveolar bone. This process involved importing the DICOM images, carefully constructing the 3D geometry, and ensuring an accurate representation of the anatomical structures in various planes.

Once the model was established, the next step was to generate a finite element mesh. The entire structure was subdivided into smaller elements connected by nodes, allowing for detailed stress analysis. The mesh consisted of 342,461 elements and 1,710,792 nodes. Boundary conditions were applied to the peripheral nodes of the alveolar region, restricting their movement in all three spatial planes (x, y, and z). This step ensured that the model behaved realistically under applied forces, replicating the constraints imposed by the surrounding structures [14,15].

As it is an FEM study, specificity of bone loss, including 0% (no bone loss), 33% bone loss, and 66% bone loss, was created on the mandibular model. Force simulations with utility arch wire and RCS arch wire were then generated. Any scenario other than those included in the criteria was excluded from the present FEM study.

Orthodontic appliances, including brackets and arch wires, were then modeled and integrated into the FEM. The brackets were designed based on the 0.022” x 0.028” slot MacLaughlin, Bennett, and Trevisi prescription. They were placed at the facial axis points of the teeth to ensure accurate simulation of clinical scenarios. The arch wires, made from 0.017” x 0.025” stainless steel, were also modeled to simulate both the utility intrusion arch and RCS arch mechanics. The interaction between these orthodontic components and the surrounding tooth structures was incorporated into the model [14,15]. To simulate the affected periodontium, three variations of bone loss were modeled: 0% (no bone loss), 33% bone loss, and 66% bone loss. These scenarios represented different levels of periodontal damage affecting the tooth-supporting bone structure. The FEM model was subjected to force simulations representing orthodontic intrusion (force magnitude: 10-20 grams per tooth (converted to Newtons: 0.098-0.196 N)) using both the utility intrusion arch wire and RCS arch wire. This allowed for the evaluation of stress distribution and displacement in the mandibular incisors under different bone loss conditions.

## Results

The results obtained from the previously described clinical scenarios, translated into a mathematical model for the mandibular four incisors, are summarized in Table 1.

**Table 1:** Comparison of stress and displacement among utility intrusion arch wire and reverse curve of Spee arch wire at various levels of bone loss

Arch wires	0% bone loss		33% bone loss		66% bone loss	
	Stress	Displacement	Stress	Displacement	Stress	Displacement
Utility intrusion arch wire	4.618E + 00	0.005	5.254E + 00	0.02	5.620E + 00	0.03
Reverse curve of Spee arch wire	3.881E + 00	0.004	4.206E + 00	0.01	4.322E + 00	0.01

As the present study is based on FEM models, the derived results can only be observed and interpreted in specific contexts. No statistical analysis is applied to the data obtained, nor is any software used to aggregate or analyze the data for comparison.

**Figure 1:**
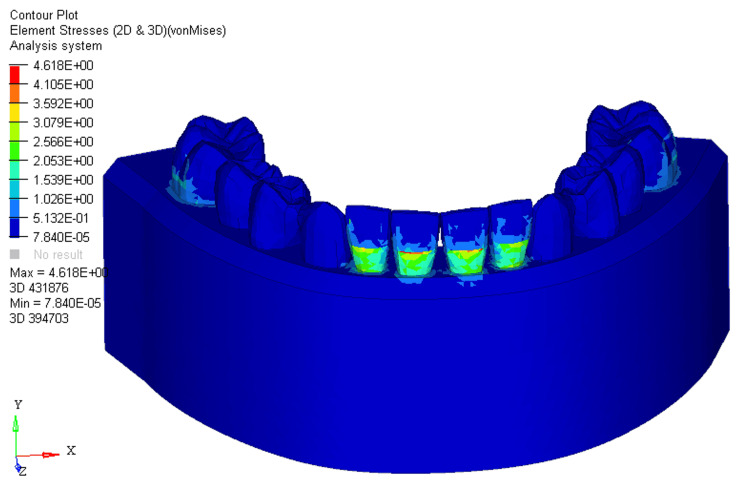
Stress distribution using utility intrusion arch wire in 0% bone loss at mandibular four incisors 2D: two dimensional, 3D: three dimensional

Under conditions of 0%, 33%, and 66% bone loss, the utility intrusion arch wire in FEM-generated models displayed distinct stress distributions, measuring 4.618E + 00, 5.254E + 00, and 5.620E + 00, respectively. The stress was distributed across the labial surface of the four mandibular incisors at different levels during the application of the utility arches. In scenarios with no bone loss, stress spanned from the middle to the cervical third on the labial surface of the crown on the mandibular four incisors (Figure 1).

**Figure 2:**
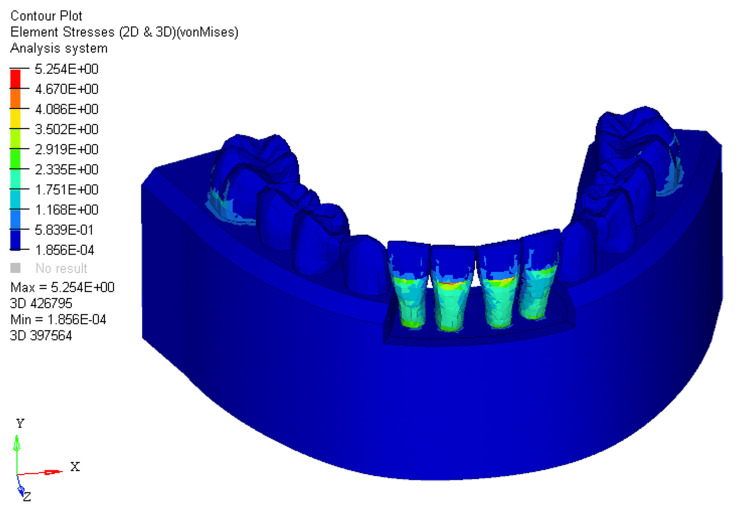
Stress distribution using utility intrusion arch wire in 33% bone loss at mandibular four incisors 2D: two dimensional, 3D: three dimensional

A shift to 33% bone loss resulted in stress moving from the middle third of the crown to the cervical third of the root on the labial surface of the mandibular four incisors (Figure 2).

**Figure 3:**
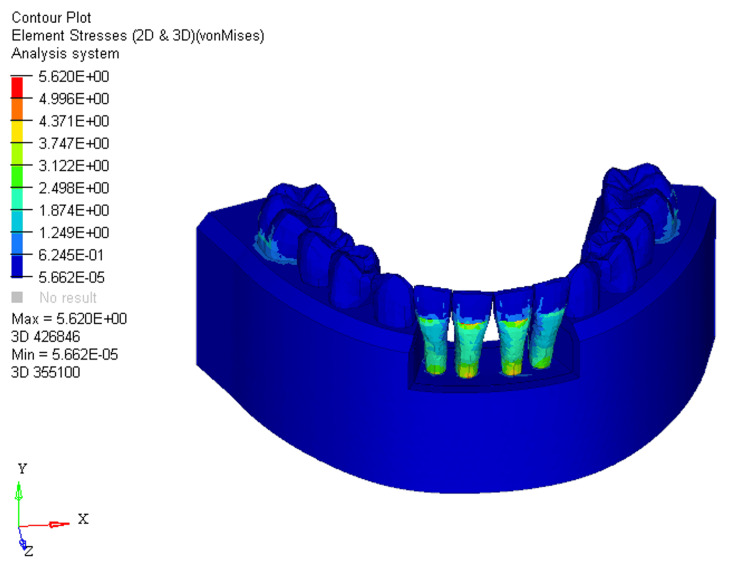
Stress distribution using utility intrusion arch wire in 66% bone loss at mandibular four incisors 2D: two dimensional, 3D: three dimensional

With 66% bone loss, stress distribution extended from the middle third of the crown to the apical third of the root on the labial surface at the mandibular four incisors (Figure 3).

**Figure 4:**
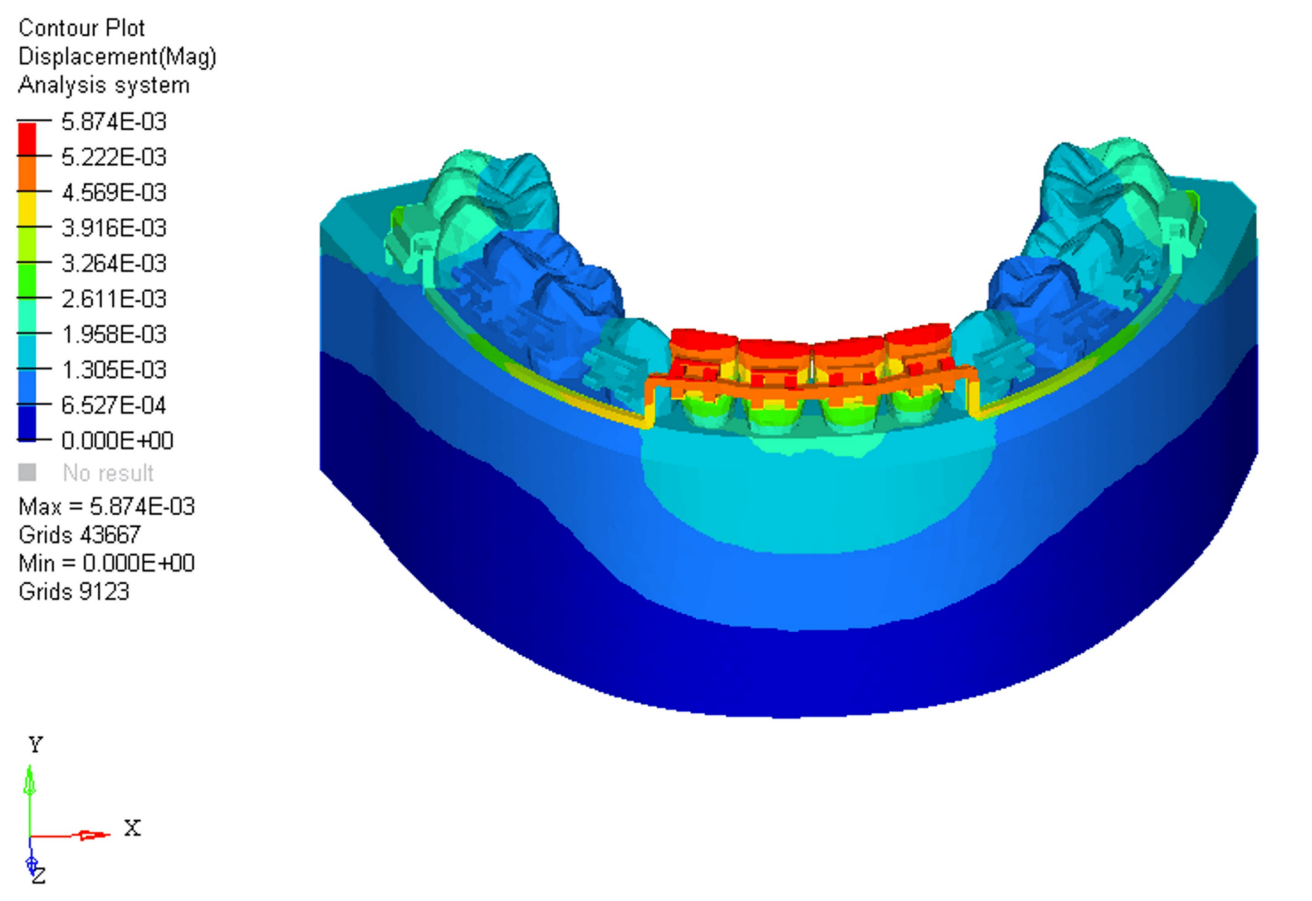
Displacement using utility intrusion arch wire in 0% bone loss at mandibular four incisors

Similarly, displacements under these conditions were measured at 0.005, 0.002, and 0.03, respectively. In the absence of bone loss, displacement was observed on the labial surface of the four mandibular incisors at the incisal level (Figure 4).

**Figure 5:**
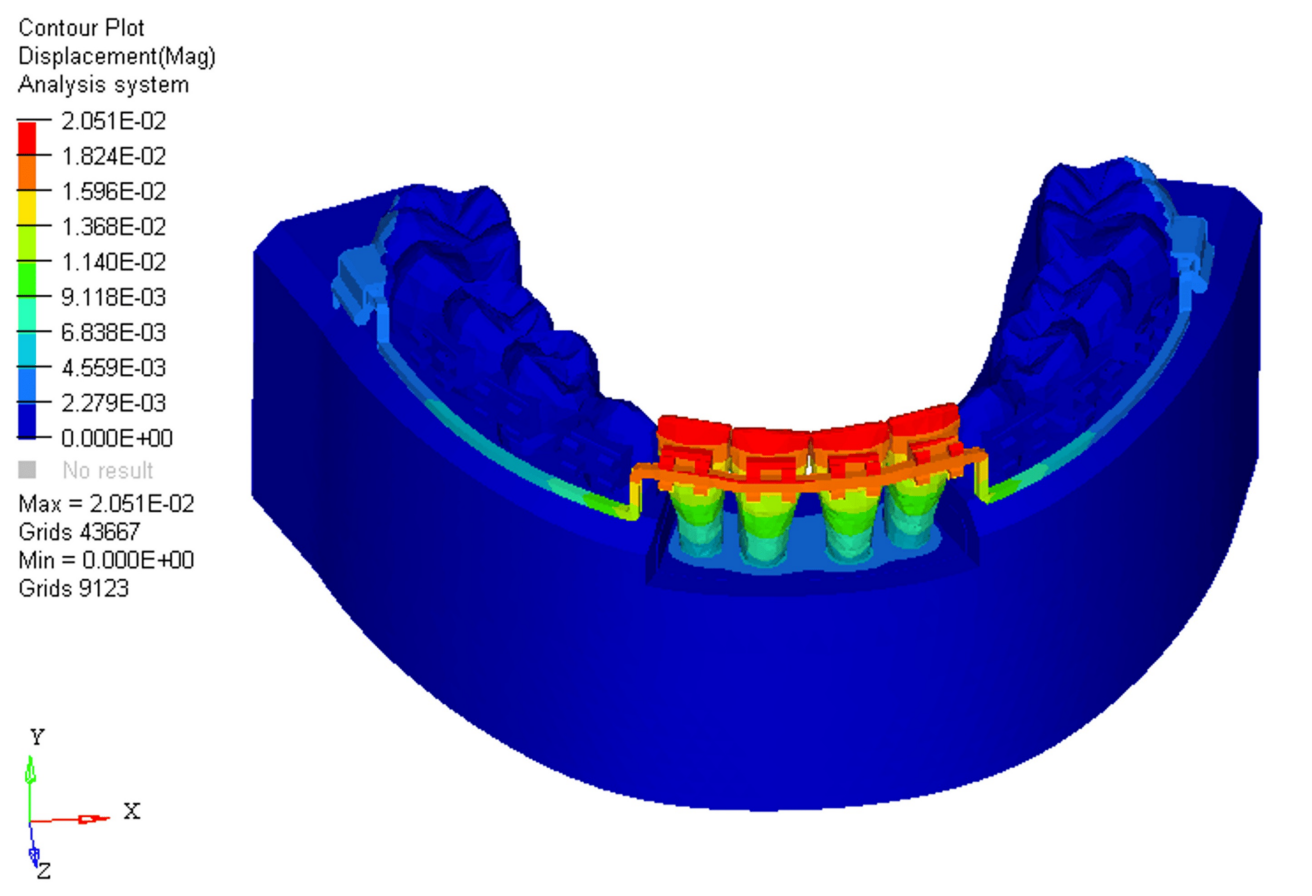
Displacement using utility intrusion arch wire in 33% bone loss at mandibular four incisors

With 33% bone loss, displacement shifted from the incisal edge to the level of the bracket slot on the four mandibular incisors (Figure 5).

**Figure 6:**
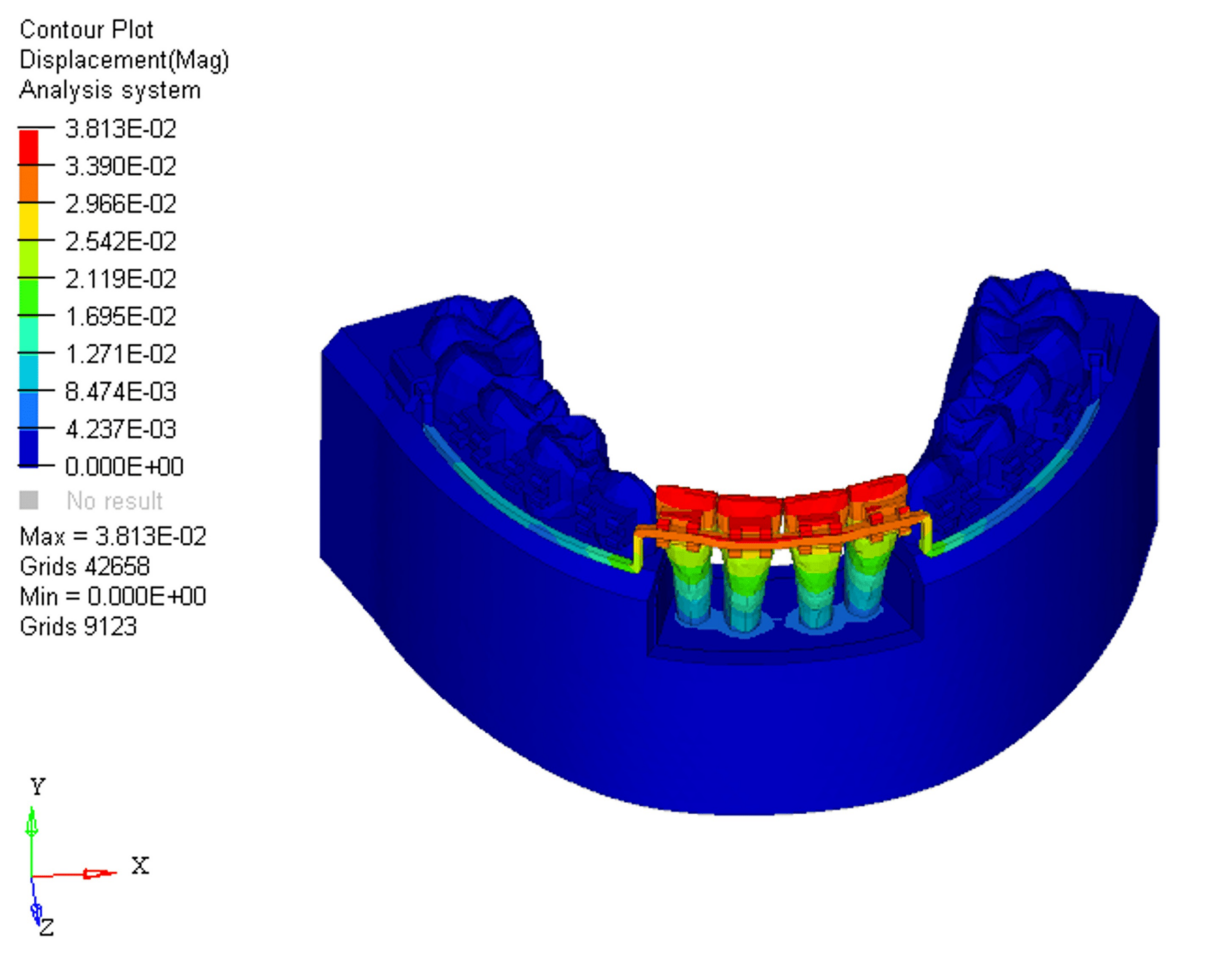
Displacement using utility intrusion arch wire in 66% bone loss at mandibular four incisors

Progressing to 66% bone loss resulted in displacement moving from the labial aspect of the incisal edge to just beneath the level of the bracket slot at the mandibular four incisors (Figure 6).

**Figure 7:**
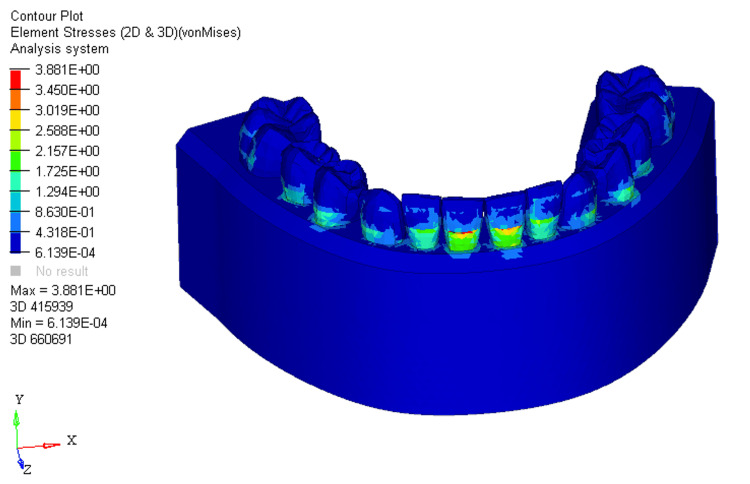
Stress distribution using reverse curve of Spee arch wire in 0% bone loss at mandibular four incisors 2D: two dimensional, 3D: three dimensional

In FEM-generated models, the application of RCS arch wires under different levels of bone loss (0%, 33%, and 66%) revealed specific stress distributions, measuring 3.881E + 00, 4.206E + 00, and 4.322E + 00, respectively. The stress was predominantly observed on the labial surface of the four mandibular incisors, with a more prominent effect on the two central incisors and minimal impact on the two lateral incisors. With no bone loss, the stress distribution covered the labial surface of the crowns from the middle to the cervical third (Figure 7).

**Figure 8:**
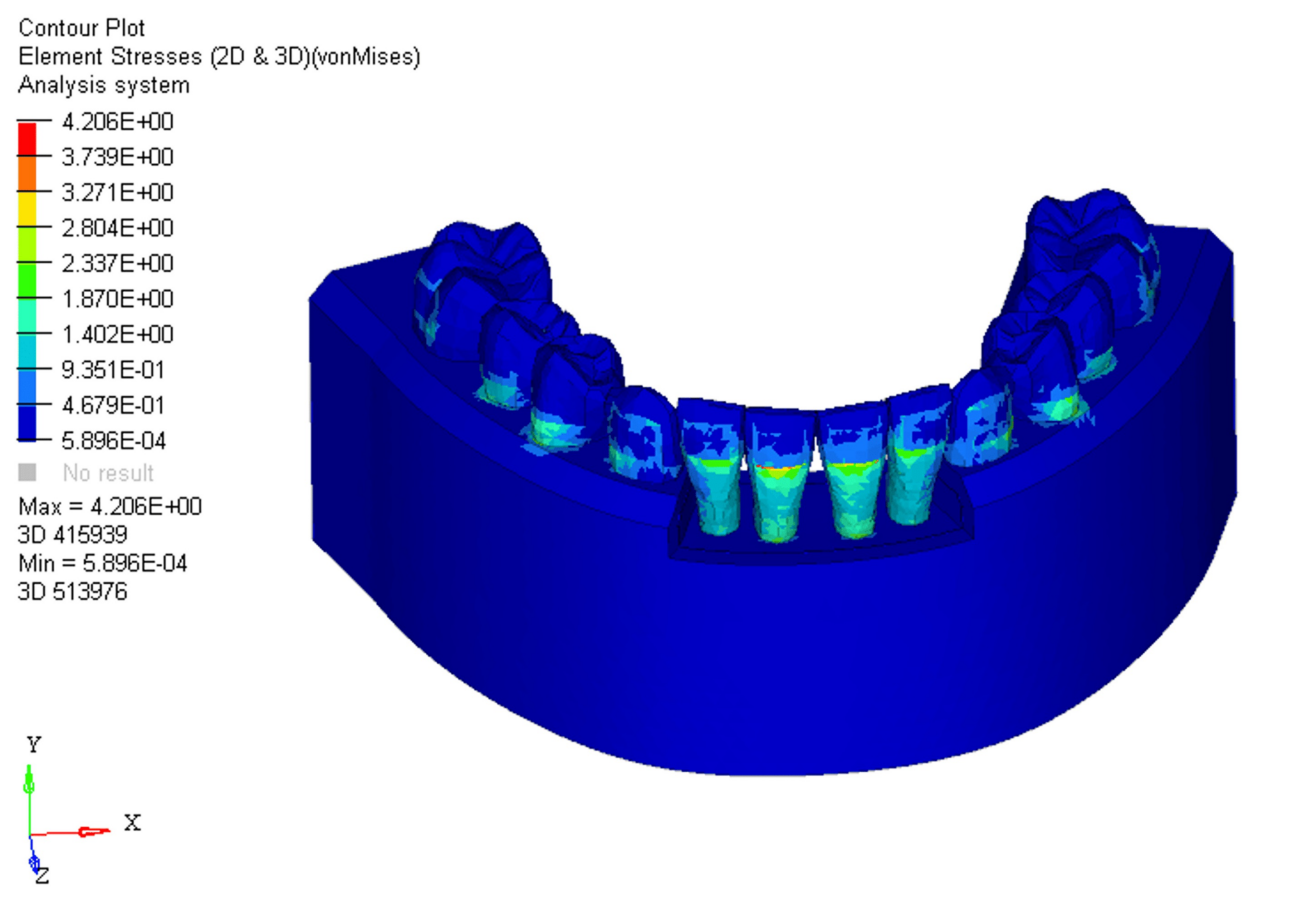
Stress distribution using reverse curve of Spee arch wire in 33% bone loss at mandibular four incisors 2D: two dimensional, 3D: three dimensional

A shift to 33% bone loss resulted in stress moving from the middle third of the crown to the cervical third of the root, primarily affecting the two mandibular central incisors (Figure 8).

**Figure 9:**
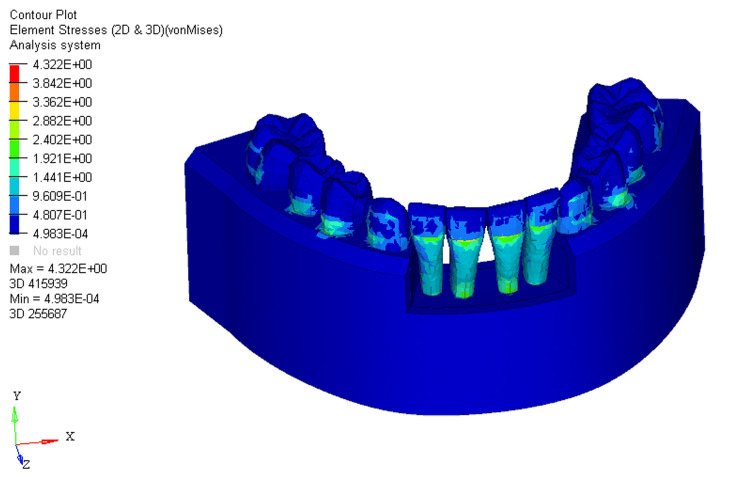
Stress distribution using reverse curve of Spee arch wire in 66% bone loss at mandibular four incisors 2D: two dimensional, 3D: three dimensional

With 66% bone loss, stress extended from the middle third of the crown to the apical third of the root, predominantly affecting the two mandibular central incisors on the labial surface (Figure 9).

**Figure 10:**
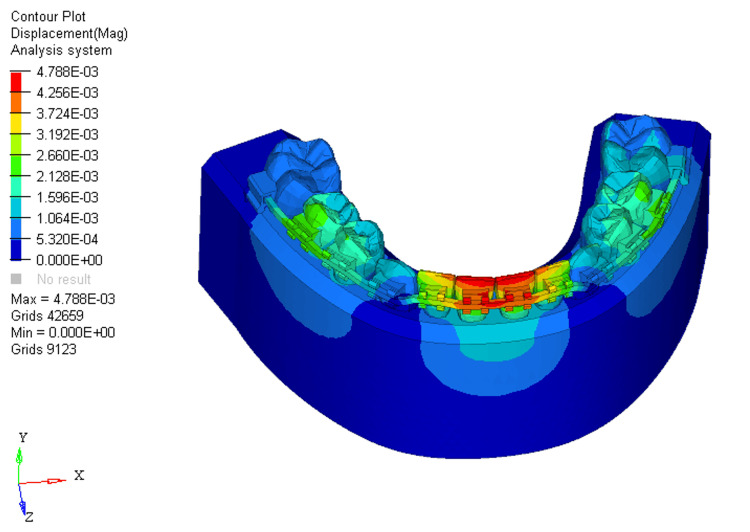
Displacement using reverse curve of Spee arch wire in 0% bone loss at mandibular four incisors

Similarly, displacements under these conditions were measured at 0.004, 0.01, and 0.01, respectively. Without bone loss, displacement primarily occurred on the labial surface of the two mandibular central incisors at the incisal level (Figure 10).

**Figure 11:**
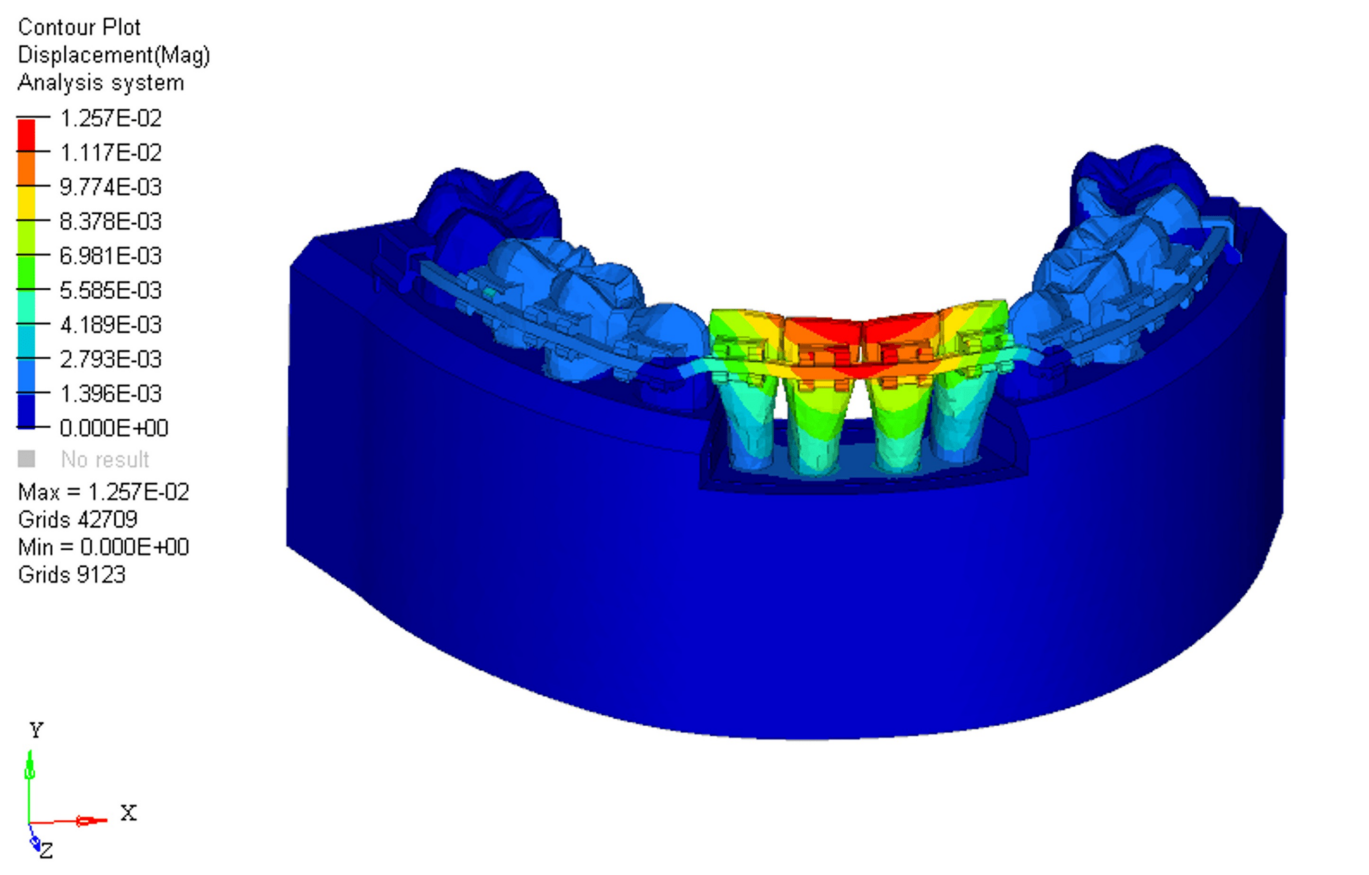
Displacement using reverse curve of Spee arch wire in 33% bone loss at mandibular four incisors

At 33% bone loss, displacement shifted from the incisal edge to the bracket slot level on the two mandibular central incisors to a greater extent, with minimal impact on the two mandibular laterals (Figure 11).

**Figure 12:**
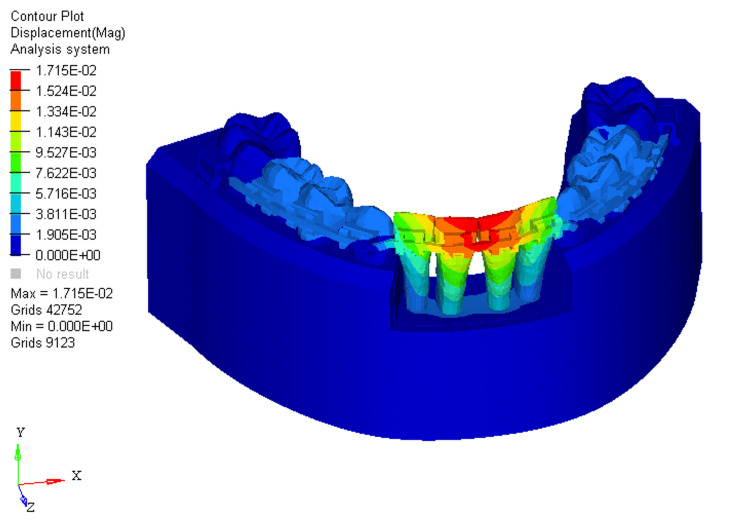
Displacement using reverse curve of Spee arch wire in 66% bone loss at mandibular four incisors

With 66% bone loss, displacement further shifted from the labial aspect of the incisal edge to just beneath the bracket slot levels at the mandibular four incisors (Figure 12).

## Discussion

With progressive bone loss, the center of resistance moves apically, and the forces acting on the crowns generate a larger moment, contributing to the progressive displacement. Intrusion, retraction, and/or uprighting of the incisors seem to be the logical solutions to the orthodontic problems of these patients, considering the causative, aesthetic, and functional aspects [16-18]. One common observation with horizontal bone loss in the lower incisors is trauma from occlusion due to extrusion of the incisors. This can be corrected orthodontically using intrusion mechanics.

The present study uses FEM-derived utility intrusion arch wires and RCS arch wires to analyze the stress distribution and displacement affecting the periodontium at different levels of bone loss (0%, 33%, and 66%) during the intrusion of the lower incisors. To standardize the study protocol and simulate bone loss at specific levels, approximations of 33% and 66% bone loss were considered to represent loss from the incisal to middle third and middle to apical third, respectively, of the mandibular incisors. The materials used in this study are consistent with previous research, which reported the following mechanical properties: tooth (Young’s modulus - 20 GPa, Poisson’s ratio - 0.30), cortical bone (Young’s modulus - 13.7 GPa, Poisson’s ratio - 0.30), spongy bone (Young’s modulus - 345 MPa, Poisson’s ratio - 0.30), and the periodontal ligament (Young’s modulus - 0.71 MPa, Poisson’s ratio - 0.30) [14,19].

In the FEM models for utility intrusion arch wires, stress distribution under the 0%, 33%, and 66% bone loss conditions was found to be 4.618E+00, 5.254E+00, and 5.620E+00, respectively. Stress was primarily distributed across the labial surface of the four mandibular incisors. As bone loss increased, the stress distribution shifted from the middle third of the crown to the apical third of the root. This shift is due to the change in the center of resistance, which moves closer to the root apex as bone loss progresses. At 0% bone loss, the stress was concentrated from the middle third of the crown to the cervical third, while at 33% bone loss, stress extended from the middle third of the crown to the cervical third of the root. With 66% bone loss, the stress shifted even further, reaching the apical third of the root on the labial surface. This change in stress distribution demonstrates how increasing bone loss alters the way forces are applied to the teeth, with more stress concentrated around the root as bone loss increases. The displacements for the utility intrusion arch wires were measured at 0.005, 0.002, and 0.03 for the 0%, 33%, and 66% bone loss conditions, respectively. With no bone loss, displacement occurred at the incisal edge of the four mandibular incisors. As bone loss increased, the displacement shifted from the incisal edge to the level of the bracket slot. At 66% bone loss, displacement occurred just beneath the bracket slot. This shift in displacement suggests that the teeth are gradually tipping labially as bone loss increases. The increased displacement, accompanied by more bone loss, indicates that the teeth are moving more, which could lead to increased tipping and misalignment if not carefully controlled.

For RCS arch wires, the stress distribution in the 0%, 33%, and 66% bone loss conditions was 3.881E+00, 4.206E+00, and 4.322E+00, respectively. The pattern of stress distribution was similar to that of the utility arch wires, with stress shifting from the middle third of the crown to the apical third of the root as bone loss increased. However, the stress levels for the RCS arch wires were generally lower than those for the utility intrusion wires across all bone loss conditions. The two mandibular central incisors showed the most stress, with the mandibular lateral incisors experiencing minimal stress in comparison. This indicates that RCS arch wires place greater stress on the mandibular central incisors than on the mandibular lateral ones, which could lead to more pronounced movement in the mandibular central incisors, especially with greater bone loss. The displacement values for the RCS arch wires were 0.004, 0.01, and 0.01 for 0%, 33%, and 66% bone loss, respectively. Similar to the utility arch wire results, the displacement primarily occurred at the labial surface of the two mandibular central incisors, and as bone loss increased, the displacement shifted from the incisal edge to the bracket slot level. However, compared to the utility arch wires, the displacement for the RCS arch wires was higher at 33% and 66% bone loss, indicating more labial tipping of the mandibular central incisors. This suggests that the RCS arch wires may exert more tipping forces on the teeth as bone loss increases, leading to a more pronounced labial displacement.

The study found an inverse relationship between stress distribution and displacement. As bone loss increased, stress distribution increased, while displacement values decreased, indicating a tendency for the teeth to tip labially. In the utility arch wire group, stress distribution was higher compared to the RCS arch wires across all levels of bone loss. However, despite the higher stress levels, the utility arch wires demonstrated better torque control, leading to less labial tipping of the mandibular incisors compared to the RCS arch wires. The displacement and tipping patterns also showed that the mandibular central incisors in the RCS group experienced more labial tipping and greater displacement than those in the utility arch wire group, indicating that RCS arch wires may exert more uncontrolled tipping forces, especially in cases of bone loss. The study showed that utility intrusion arch wires performed better in terms of torque control and reduced labial tipping of the mandibular incisors when compared to RCS arch wires. Even though utility arches produced higher stress distributions, they allowed for more controlled tooth movement, which is crucial when managing teeth with reduced periodontal support due to bone loss. The results highlight the importance of selecting the appropriate orthodontic mechanics in cases of periodontal disease and bone loss, with utility arch wires providing more precise control over tooth movement and less risk of labial tipping.

Limitations

The FEM was derived from CT scan data, allowing for the accurate transformation of morphology into the model and thereby replicating the clinical situation through a mathematical model. However, it was assumed that all the structures in the model have linear, isotropic material properties, which is not valid in real-life conditions.

Future directions

Future research could incorporate different intrusion arch mechanics and variations of bone loss to assess their impact under specific applied forces in FEM models. Additionally, validating and comparing the treatment outcomes of simulated FEM models in clinical scenarios could further contribute to improving the clinical application of orthodontic treatments.

## Conclusions

Increased bone loss at the mandibular incisors correlates with increased stress distribution and diminished displacement values, indicative of labial tipping. The utility intrusion arch wire demonstrated higher stress distribution compared to the RCS arch wire across various degrees of bone loss (0%, 33%, and 66%). Notably, the RCS arch wire group exhibits evident labial tipping of the two mandibular central incisors. In contrast, the utility intrusion arch wire group consistently shows the least displacement features at all four mandibular incisors in the generated FEMs, even under varied conditions of bone loss.
